# Morpho-functional characterization of the systemic venous pole of the reptile heart

**DOI:** 10.1038/s41598-017-06291-z

**Published:** 2017-07-27

**Authors:** Bjarke Jensen, Signe Vesterskov, Bastiaan J. Boukens, Jan M. Nielsen, Antoon F. M. Moorman, Vincent M. Christoffels, Tobias Wang

**Affiliations:** 1 Department of Medical Biology, Academic Medical Center, University of Amsterdam, Amsterdam, The Netherlands; 2 Department of Bioscience, Zoophysiology, Aarhus University, Aarhus, Denmark; 3 Department of Cardiology, Institute of Clinical Medicine, Aarhus University Hospital, Skejby Aarhus, Denmark

## Abstract

Mammals evolved from reptile-like ancestors, and while the mammalian heart is driven by a distinct sinus node, a sinus node is not apparent in reptiles. We characterized the myocardial systemic venous pole, the sinus venosus, in reptiles to identify the dominant pacemaker and to assess whether the sinus venosus remodels and adopts an atrium-like phenotype as observed in mammals. *Anolis* lizards had an extensive sinus venosus of myocardium expressing *Tbx18*. A small sub-population of cells encircling the sinuatrial junction expressed *Isl1*, *Bmp2*, *Tbx3*, and *Hcn4*, homologues of genes marking the mammalian sinus node. Electrical mapping showed that hearts of *Anolis* lizards and *Python* snakes were driven from the sinuatrial junction. The electrical impulse was delayed between the sinus venosus and the right atrium, allowing the sinus venosus to contract and aid right atrial filling. In proximity of the systemic veins, the *Anolis* sinus venosus expressed markers of the atrial phenotype *Nkx2-5* and *Gja5*. In conclusion, the reptile heart is driven by a pacemaker region with an expression signature similar to that of the immature sinus node of mammals. Unlike mammals, reptiles maintain a sinuatrial delay of the impulse, allowing the partly atrialized sinus venosus to function as a chamber.

## Introduction

The heartbeat of ectothermic vertebrates is driven by a pacemaker within the sinus venosus, the myocardium upstream of the right atrium^1^. This pacemaker is not anatomically identifiable and electrophysiological studies suggest that the position of the pacemaker within the sinus venosus may differ between species^2–5^. In mammals and birds, the pacemaker resides within an anatomically distinguishable structure, the sinus node, positioned at the sinuatrial junction^6, 7^. It drives heart rates that are several fold higher than in similarly sized ectotherms^8^. For reptiles it is unclear where the pacemaker is located and how the sinus myocardium of reptiles compares to the caval vein myocardium of mammals with regards to gene expression profile, electrical activation, and capacity for contraction^9^. It is therefore difficult to assess, firstly, to what extent the sinus node of endotherms is derived from resembling tissues in ectotherms and secondly, whether the sinus venosus in general has been remodelled during the evolution from a state of ectothermy and low heart rates to endothermy and high heart rates.

In placental mammals, the sinus node and the myocardium of the systemic caval veins derive from the embryonic sinus venosus myocardium. The embryonic sinus myocardium can be identified by the expression of the transcription factor Tbx18, while the atrial myocardium can be identified by the expression of the transcription factor Nkx2-5, which is absent in the sinus myocardium^10^. During fetal development, the mammalian sinus myocardium except for parts of the sinus node gains expression of Nkx2-5 and downstream target genes including *Gja5* (Cx40)^11, 12^. These changes are referred to as atrialization of the sinus venosus^10^. The atrialization also involves widening of the sinuatrial junction, whereby the two leaflets of the myocardial sinuatrial valve become far apart and the sinuatrial junction is then, effectively, unguarded^13^.

In mammals, chicken and zebrafish, sinus myocardium expresses the transcription factors Shox2 and Tbx18 and the ligand Bmp4^10, 14, 15^. Shox2 is a transcriptional regulator of Isl1^16^, which together with Tbx3 controls the identity of the sinus nodal cells^17, 18^. Transcription factors Isl1 and Tbx3 are only expressed in the sinus node part of the sinus myocardium with greatest rate of spontaneous depolarization^4, 19, 20^. Pacemaker activity is strongly influenced by the so-called funny current carried by the family of hyperpolarization-activated cyclic nucleotide-gated K^+^-channels, including Hcn4^4, 18, 21^.

We undertook an anatomical, molecular, electrophysiological and pharmacological investigation of the sinus myocardium in two commonly investigated reptiles, the *Anolis* lizard and the python. The heart of the *Anolis* lizard may be seen as representative of most hearts of non-crocodilian reptiles^9^. Conversely, the ventricle of pythons show greater resemblance to the mammalian ventricles by having an almost full ventricular septum that allows for high systemic blood pressure, while maintaining low pulmonary blood pressures^22^. Our results show that the reptilian sinuatrial junction function as the dominant pacemaker of the heart and has a molecular phenotype comparable to those of embryonic mammals and chicken. The sinuatrial junction exhibits a delay in impulse propagation that enables time for the so-called caval vein myocardium, or sinus venosus, to aid right atrial filling. Myocardium of the sinus venosus propagates the electrical current fast and its distal parts have an atrialized molecular phenotype. Our data suggest that in the evolution of mammals the sinus node developed from a large ring-like domain of myocardium with nodal characteristics, the sinuatrial delay was lost, and the sinus venosus myocardium became extensively atrialized.

## Results

### Molecular identification of the pacemaker of the reptile heart at the sinuatrial junction

**Figure 1:**
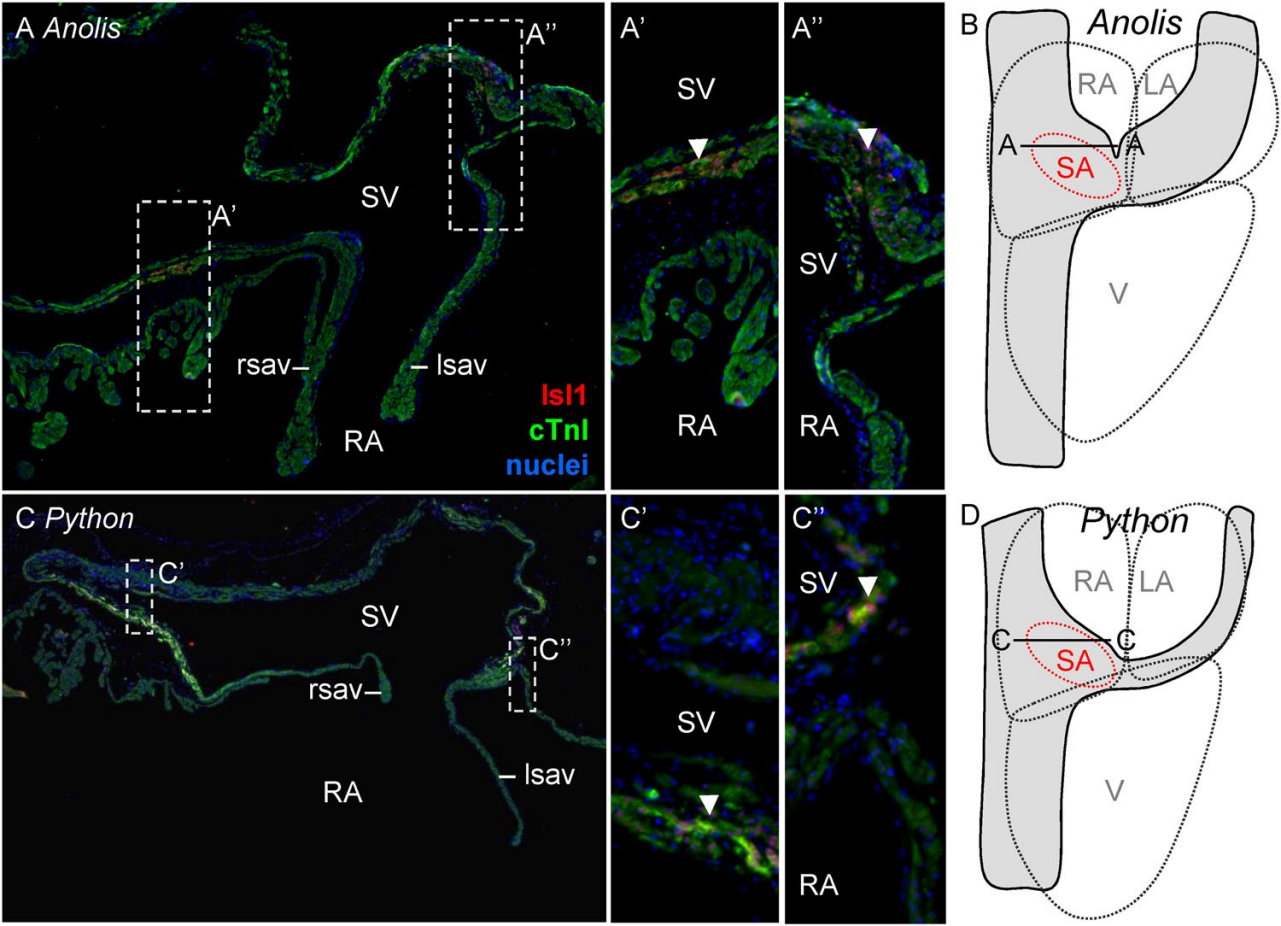
Identification of the putative dominant pacemaker by Isl1 detection. (**A**) Histological section of sinuatrial junction of the adult *Anolis equestrie*, showing the left and right leaflet of the sinuatrial valve (lsav and rsav, respectively). Isl1 in myocardial nuclei is expressed (arrow heads) immediately upstream of the sinuatrial valve in a ring-like domain. (**B**) This cartoon indicates the sectioning plane (A-A) relative to the sinuatrial junction (SA) and whole heart. The outline of the sinus venosus is indicated in grey. (**C**,**D**) Isl1 in the adult *Python regius* is expressed as in *Anolis*. LA, left atrium; RA, right atrium; SV, sinus venosus; V, ventricle.

**Figure 2:**
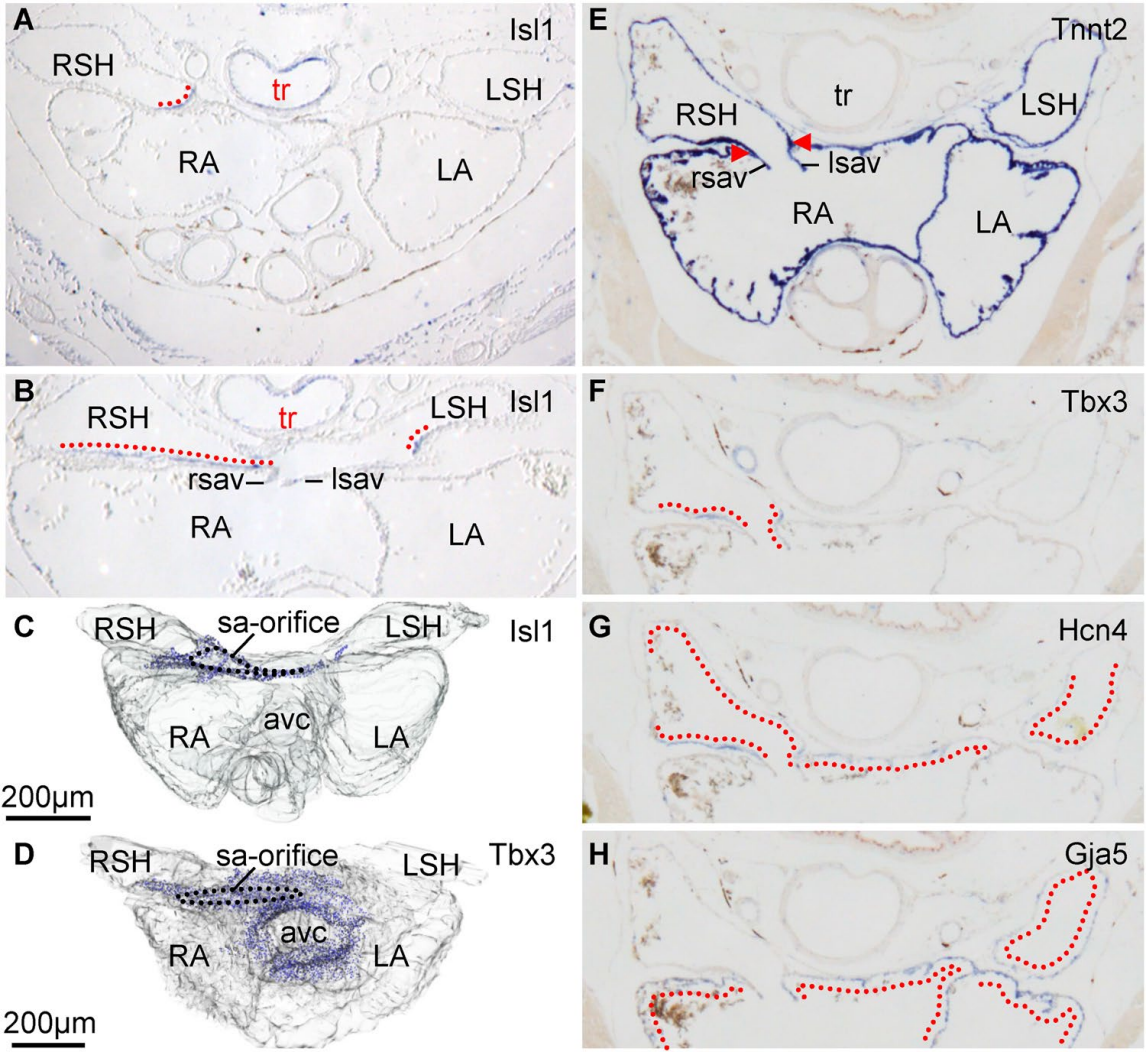
The sinuatrial junction of reptiles has the phenotype of the mammalian sinus node. (**A**,**B**) In the near-hatching *Anolis sagrei, Isl1* was detected cranial to the sinuatrial junction (**A**), where the sinus node is found in mammals, at the base of the sinuatrial valve and in the most proximal part of the left sinus horn (**B**). (**C**,**D**) The domain of *Isl1* (**C**) overlaps with the domain of *Tbx3* (**D**). (**E–H**) The anole sinus myocardium proximal to the sinuatrial junction expresses *Tbx3* and *Hcn4*, but not *Gja5*. avc, atrioventricular canal; lsav, left leaflet of the sinuatrial valve; LA, left atrium; LSH, left sinus horn; RA, right atrium; rsav, right leaflet of the sinuatrial valve; RSH, right sinus horn; sa-orifice, sinuatrial-orifice; tr, trachea.

**Figure 3:**
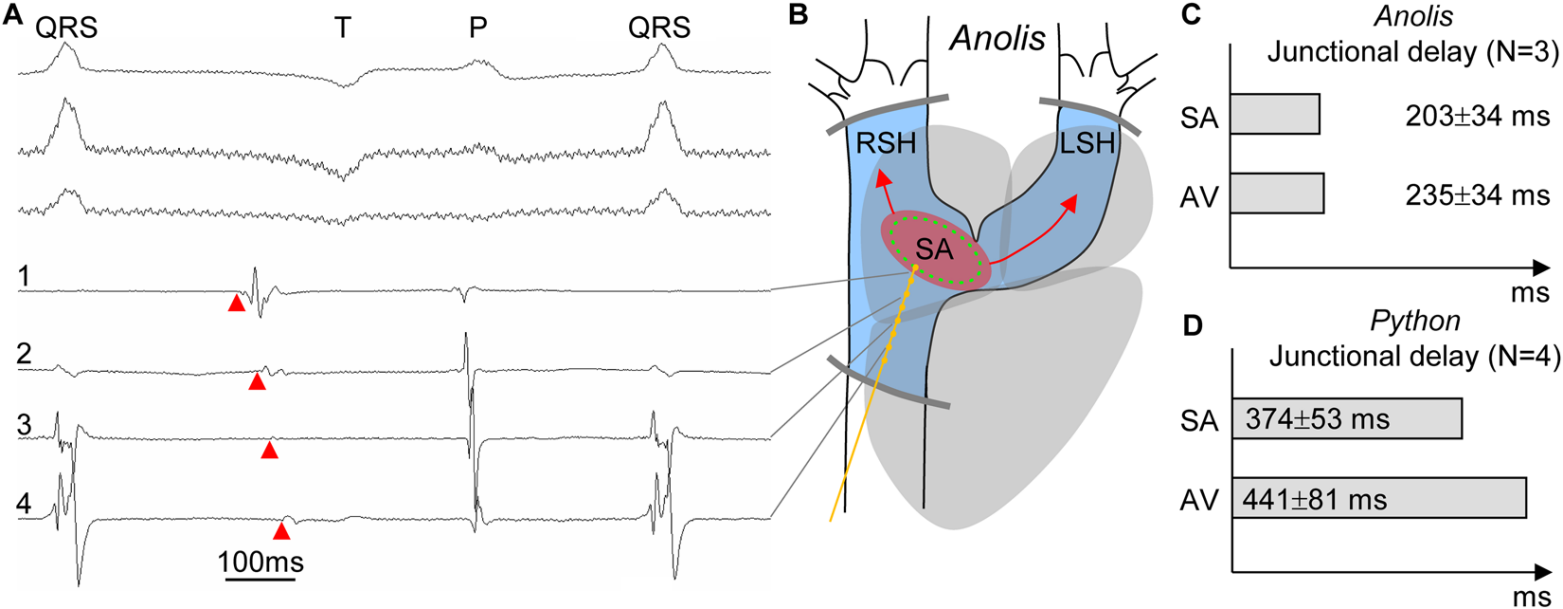
The sinuatrial junctional area harbors the dominant pacemaker, has a delay, and the sinus venosus is activated retrograde in both *Anolis* and *Python*. (**A**) Original traces of three-lead surface ECG (upper part of the panel) and local recordings from 4 bi-polar electrodes in *Anolis equestrie* (traces 1 to 4 in lower part of the panel). Electrode 1 is in the sinuatrial junctional area, whereas electrode 4 is the furthest into the posterior sinus horn. Deflections in all 4 electrodes can be seen to align with the P- and QRS-waves of the ECGs and these deflections are considered remote activity. The deflections indicated by red arrowheads occur prior to the deflections aligned with the P-wave and are therefore considered local electrograms of the sinus venosus. The earliest deflection is in electrode 1 and the latest in electrode 4, thereby showing dominant pacemaker activity in the sinuatrial junctional area and retrograde activation of the posterior sinus horn. In electrode 1, the interval between the early deflection (red arrowhead) and the deflection aligned with the P-wave constitutes the substantial sinu-atrial delay. (**B**) All sinus horns are activated retrograde from the sinu-atrial junctional area and the activation front stops at the pericardial reflection. (**C**,**D**) Within species, the sinu-atrial (SA) and atrioventricular (AV) delays are of similar duration. LSH, left sinus horn; RSH, right sinus horn.

In all investigated embryonic and adult reptiles, myocardial expression of the transcription factor Isl1, which is expressed in the developing sinus node cells in mammals, constituted a ring-like domain in the sinus myocardium immediately upstream of the right atrium (Fig. 1, Fig. 1S). The border of the sinus venosus and right atrium was guarded by a bicuspid myocardial valve in all reptilian species (Fig. 1, 1S). The sinus venosus myocardium, including the Isl1-positive domain, was rich in collagen, but there was no insulated (sinus) node (Fig. 2S). The sinus myocardium was generally thickest at the base of the sinuatrial valve, and thicker than the atrial wall. This was particularly evident in *Anolis* where the cranial part of the sinuatrial junction received a relatively large artery (Figs 2S and 3S).

Analysis of gene expression was predominantly performed in *Anolis* at different stages of development. The region of the sinus venosus that expressed *Isl1* also expressed *Tbx3* and *Bmp2* (Fig. 2A–D, 4S). Between *Anolis* stages 7 and 19, the *Isl1* domain only grew 70% while the remaining sinus venosus myocardium grew 530% (N = 2). *Hcn4* was broadly expressed in the sinus venosus and atria of the lizard, but expression was greatest in the region of the *Isl1* expression domain (Fig. 2E–G). We did not detect *Gja5* (Cx40) in the *Isl1* expression domain and in the sinus myocardium proximal to the right atrium (Fig. 2H). We also investigated the sinuatrial junction of an embryonic American alligator and again found rich expression of *Tbx3* in the sinus myocardium most proximal to the sinuatrial junction (Fig. 5S). *Gja5* and *Scn5a* were absent where *Tbx3* was present (Fig. 5S).

### The dominant pacemaker of the reptile heart is in the sinuatrial junction

Electrophysiology was exclusively done in adult *Anolis* and *Python*. The activation front of the sinus venosus always propagated away from the sinuatrial junction (Fig. 3). Electrical propagation was slower in the sinus venosus than in the atria (0.18 ± 0.11 vs 0.76 ± 0.58 m/s, N = 3 in *Anolis*). The electrical current propagated distally into all three sinus horns, but did not travel beyond the pericardial reflection (Fig. 3).

### The sinuatrial junction of the reptile heart exhibits a substantial delay

There was always a sinuatrial delay, which had approximately the same duration as the subsequent atrioventricular delay (Fig. 3). Sinuatrial and atrioventricular delays where longer in the pythons than in *Anolis* (Fig. 3C,D) and they had lower heart rates than the *Anolis* (approximately 40 beats per minute, N = 2 versus 75 ± 5 beats per minute, N = 3). In one *Python*, we stimulated the sinus venosus at incremental frequencies and found the sinuatrial junction to exhibit decremental conduction, i.e. Wenckebach phenomenon, at 1.5 Hz. Similarly, the atrioventricular canal also exhibited Wenckebach phenomenon, also at 1.5 Hz, when stimulated from the atria.

To gain better spatial resolution we used optical mapping of the sinuatrial region of the excised heart of the *Anolis* (Fig. 6S). However, in the excised and thus denervated heart, the dominant pacemaker was shifted away from the sinuatrial region and towards the posterior sinus horn (Fig. 6S).

### The sinus venosus of the reptile heart has a chamber-like phenotype

**Figure 4:**
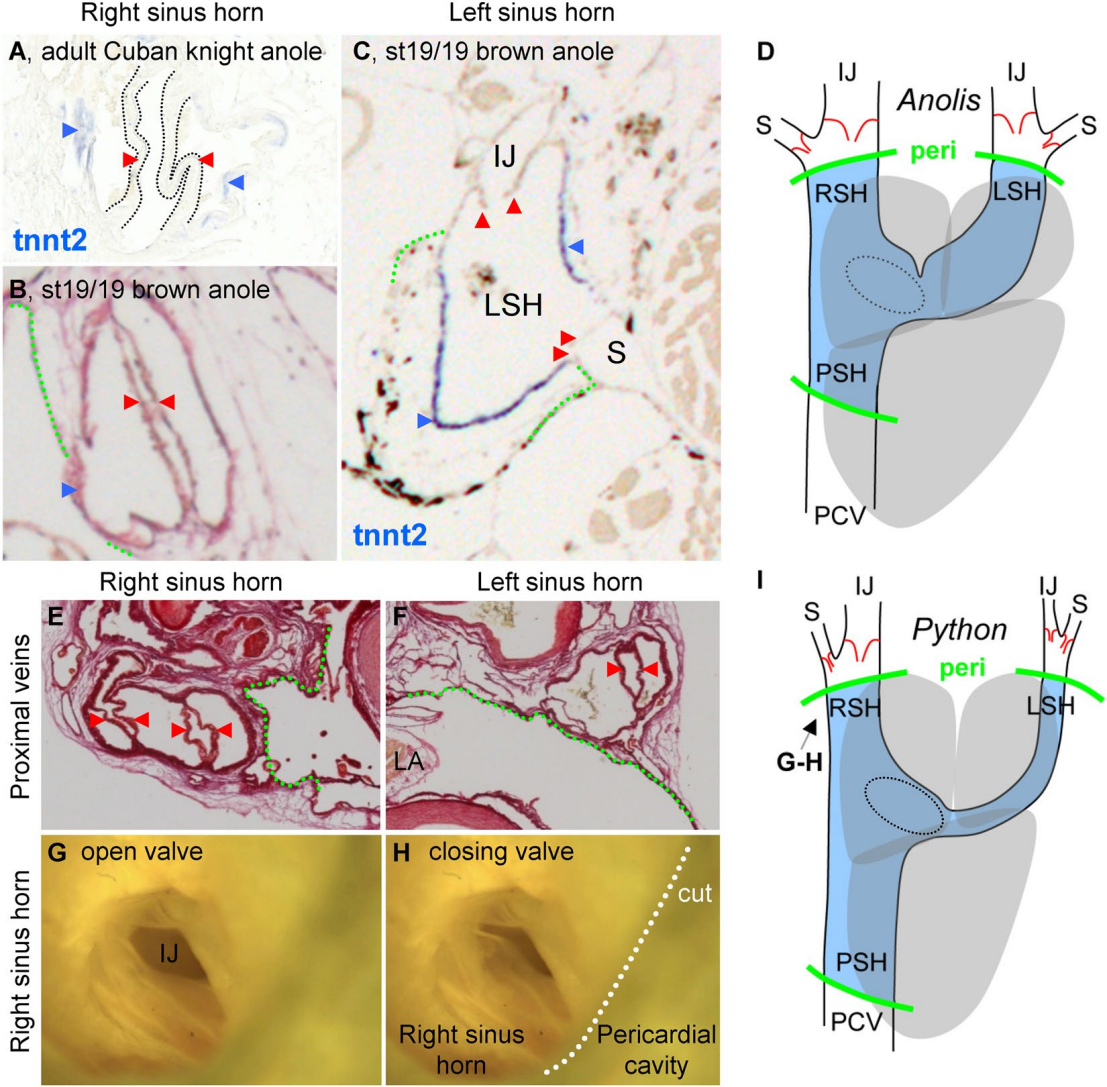
Venous valves guard the entrance to the anterior sinus horns. (**A**–**D**) Venous valves (red arrow heads) are found immediately upstream of the left and right sinus horns in *Anolis*, just outside the pericardial cavity (green broken line), but they are not found between the heart and liver. (**E**–**I**) In *Python*, the configuration of venous valves are like in *Anolis*. In (**G–H**) the leaflets of the valve between the right sinus horn and the right internal jugular vein has been spread apart (**G**) and allow to relax toward the closed state (**H**). In (**I**), the arrow indicates the angle of inspection in (**G**,**H**). IJ, internal jugular vein; LA, left atrium; LSH, left sinus horn; RA, right atrium; PCV, posterior caval vein; peri, pericardium; PSH, posterior sinus horn; RSH, right sinus horn; S, subclavian vein.

**Figure 5:**
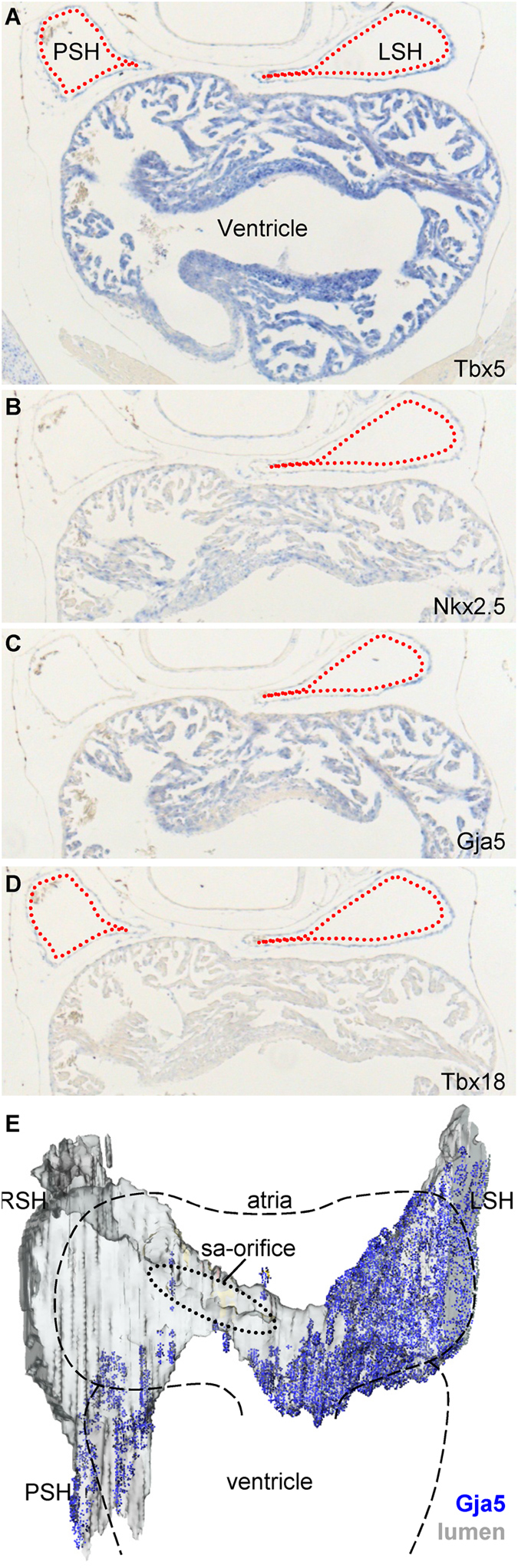
Distal parts of the sinus venosus of *Anolis sagrei* have the molecular phenotype of a chamber. (**A**,**B**) *Tbx5* is expressed in the entire sinus venosus myocardium (**A**), whereas *Nkx2.5* is expressed in the distal parts only (**B**). (**C**) Expression of *Gja5* co-localizes with *Nkx2.5*. (**D**) The sinus myocardium is distinct from the atria and ventricle by expressing *Tbx18*. (**E**) Overview of *Gja5* expression in a near-hatching *Anolis sagrei*. LSH, left sinus horn; PSH, posterior sinus horn; RSH, right sinus horn; sa, sinuatrial; V, ventricle.

The observed retrograde activation of the sinus venosus suggests that the sinus venosus functions as a chamber. In both *Anolis* and *Python* we found bicuspid venous valves immediately distal to the anterior sinus horns at the orifices of the internal jugular and subclavian veins. Consistent with generation of pressure within the sinus venosus, the free margins of the valvular leaflets pointed towards the heart (Fig. 4). No valves were found posterior to the sinuatrial junction (we investigated as far posterior as the liver). In near-hatching *Anolis*, distal parts of the sinus venosus, and the atria in entirety, expressed *Gja5*, *Nkx2.5* and *Tbx5* (Fig. 5). However, the myocardium of the sinus venosus was distinct from the atria by expression of *Tbx18* (Fig. 5D) and absence of *Tbx20* (Fig. 7S). While both atria and the ventricle were trabeculated, we never observed trabeculations in the sinus venosus. *In ovo* growth of the sinus venosus in *Anolis* paralleled that of the atria and ventricle by an increment of more than 500% from stages 7 to 19. By stage 19, just before hatching, the sinus venosus enclosed a volume approximately equal to the volume of the right atrium.

During development of *Anolis*, the left and right sinus horns expressed the myocardial marker *tnnt2* and contract from their first appearance in embryogenesis (Fig. 8S, supplementary video). A small myocardial ridge was seen to develop between the left and right sinus horns in the dorsal wall of the sinus, the so-called sinus septum. It expressed *Tbx3* and appeared to be a myocardialized part of the dorsal mesenchymal protrusion (*Isl1*-positive intrapericardial mesenchyme^23^).

**Figure 6:**
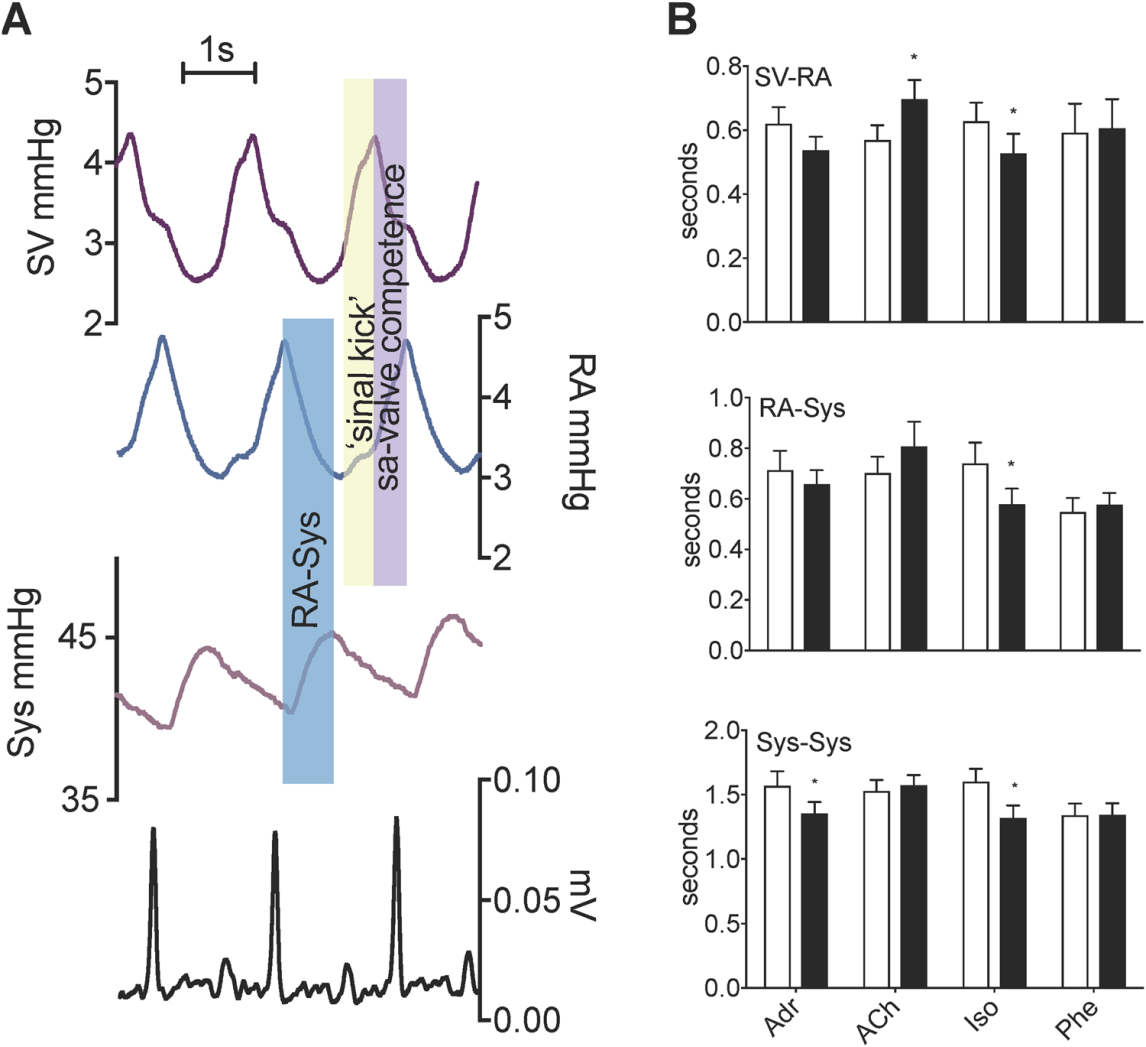
The sinus venosus of *Python* functions as a chamber. (**A**) The sinus venosus beats prior to but in synchrony with the atria and ventricle. (**B**) Blood pressures showing similar responses in the sinus venosus, right atrium, and ventricle to the β-adrenergic agonist isoproterenol. Adr; adrenaline (10 μmol L^−1^), ACh; Acetylcholine (1 μmol L^−1^), Iso; isoproterenol (100 μmol L^−1^), Phe; phenylephrine (30 μmol L^−1^). N = 6. Asterisk indicates a significant difference from control value (P < 0.05, two-tailed, paired t-test).

In adult anaesthetized *Python*, blood pressure increased earlier in the sinus venosus than in the right atrium, consistent with the pattern of electrical activation (Fig. 6). The blood pressure of the right atrium increased in two steps. The first step coincided with the increment of blood pressure in the sinus venosus, consistent with a ‘sinal kick’ contributing to atrial filling (Fig. 6A). The second and greater increment of right atrial pressure coincided with a decrease in sinus pressure (diastole of the sinus) consistent with a competent sinuatrial valve during atrial systole (Fig. 6A). Both chronotropic and dromotropic responses to β-adrenergic and muscarinic stimulation were observed, as intra-arterial injections of adrenalin and isoproterenol caused an increase in heart rate and a shortening of the time between peak-pressures of the sinus venosus and the right atrium (Fig. 6B). Acetylcholine increased the time between peak-pressures of the sinus venosus and the right atrium together with a decrease in heart rate (Fig. 6B). Inotropic responses to β-adrenergic stimulation was found in strips of sinus venosus, as adrenalin and isoproterenol elicited an increment in active tension whereas acetylcholine elicited a decrement in active tension (Fig. 9S). Strips of sinus venosus and atria produced twitches only and tetanus could not be induced.

## Discussion

**Figure 7:**
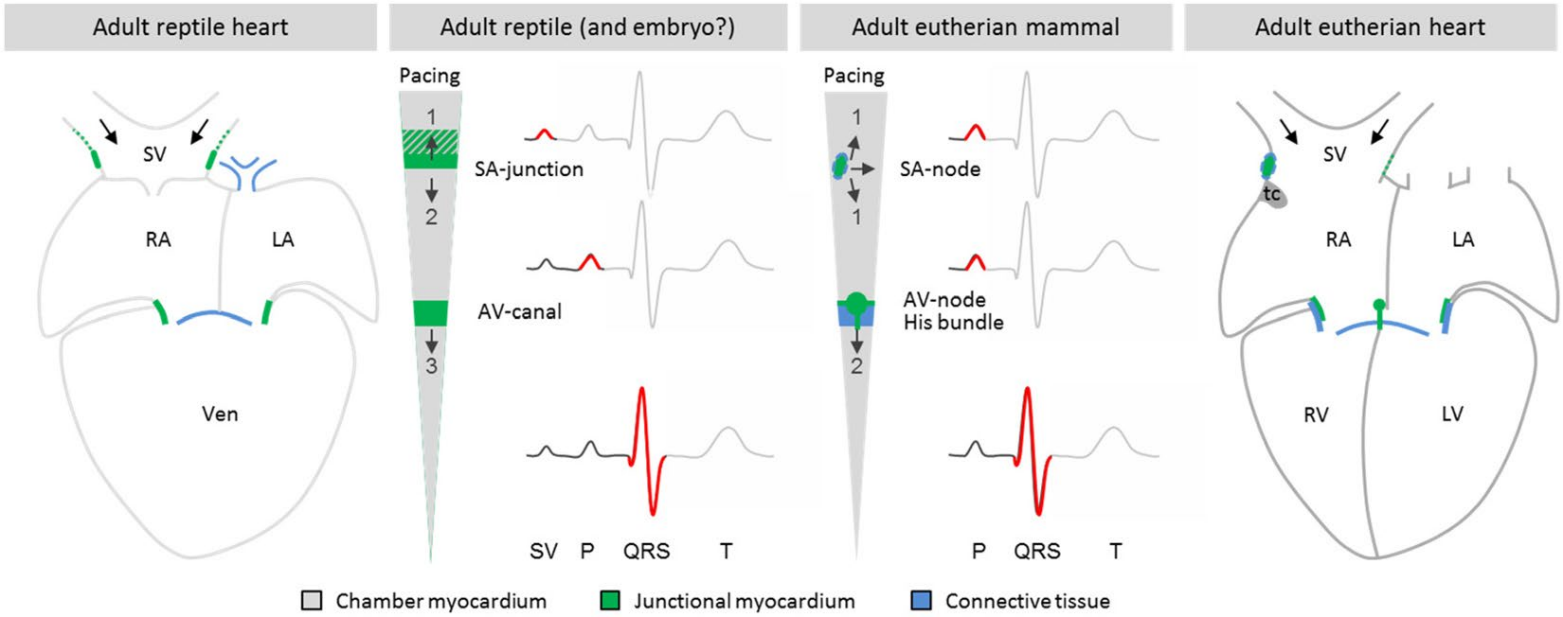
The sinuatrial junction of reptiles and mammals. Reptiles have a sinuatrial junction which harbors the dominant pacemaker and establishes a delay between the electrical activation of the sinus venosus and the atria. This enables the sinus venosus to function as a chamber that aids the filling of the right atrium. In eutherian mammals, the sinuatrial junction is remodeled and the sinuatrial delay is lost as revealed on the ECG by the absence of a sinus venosus wave. The sinus venosus of embryonic mammals may function much like in reptiles, and the mammalian sinus node develop from tissues with a similar phenotype as the reptilian sinuatrial junction.

In mammals and birds, the dominant pacemaker is situated in a sinus node on the sinuatrial junction^6, 7, 24^. In the zebrafish and trout, the dominant pacemaker is situated in a ring-like domain that constitute the sinus part of the sinuatrial junction. Modern bony fishes like zebra fish, however, are unusual in having very little if any myocardium in the sinus venosus^4, 25^. Conversely, in reptiles the sinus venosus is populated by myocardium. As the dominant pacemaker is always at the intake of the heart in early developmental stages^26^, a pacemaker could be anticipated within any, or all, of the sinus horns of the reptile heart. Yet, we show the dominant pacemaker remains in a ring-like domain in the immediate vicinity of the sinuatrial junction (Fig. 7). This observation is in line with those in previous studies using body surface electrocardiography in squamate reptiles and sharp electrodes on the cranial sinuatrial junction of the American alligator^27, 28^. Although we could not identify a nodal structure in the sinuatrial junction, there is a relatively large coronary artery that we hypothesize is homologous to the sinuatrial nodal artery of the mammalian heart^29^, and the sinus myocardium is thick in the vicinity of the artery, even when compared to the atrial wall. Interestingly, it is thought that the dominant pacemaker has to be insulated to generate sufficient current to drive the much larger atrial mass^26, 30, 31^. However, the observed absence of distinct insulation by connective tissues around the dominant pacemaker region, marked by *Tbx3* and Isl1, indicates that at least in the ectothermic vertebrates insulation by connective tissues is not required for pacemaker function.

In reptiles, the sinuatrial and atrioventricular delays are of fairly similar duration, as previously reported^27^. The sinuatrial and atrioventricular junctions are similar by expression of *Bmp2* and *Tbx3*, components of a transcriptional network suppressing chamber development. Accordingly, both junctions appear without *Gja5* (Cx40), a marker of chamber development that is associated with fast propagation of the electrical impulse^32^. The sinuatrial and atrioventricular junctions exhibit the Wenckebach phenomenon, as shown here for the ball python and previously for other species of snake and a freshwater turtle^33, 34^, a phenomenon shared with the sinus node and atrioventricular node of the mammalian heart. We found a tiny population of sinus myocardium between the sinuatrial valve and the Isl1-positive domain. Positioned such, this myocardium appears to participate in the sinuatrial delay. The findings of positive and negative dromotropic effects in response to adrenergic and muscarinic agonists respectively, suggests that, not only is nodal-like tissue present in reptiles, it is also subject to a similar type of innervation, as is the case for the AV-nodal tissue.

In contrast to other cardiac chambers of the reptile heart, the sinus venosus myocardium is activated retrograde, towards the veins rather than towards the arterial pole (Fig. 7), as previously reported for the left sinus horn in snakes^35^. The mammalian caval vein myocardium is also activated retrograde (recently reviewed in^5^). Mammals generally have activation well into the superior caval veins and even the azygos vein^36, 37^, although it is controversial to what extend the mouse caval vein myocardium is activated^38^. Most mammals maintain a left superior caval vein^5, 39^, but in human it usually regresses during development. When the human left superior caval vein fails to regress, it is also activated retrograde^5^. As the sinus venosus of reptiles consists of three vessels, essentially configured like a Y, first activation in any horn (myocardial sleeve) will result in retrograde activation of the two other sinus horns. With the cardiac impulse originating from the sinuatrial junction, the activation front of the three sinus horns will travel in roughly opposite directions. The opposite directions of the propagating impulse together with the small mass of the sinus myocardium may explain why the activation of the sinus venosus is rarely detected on the body surface ECG^5, 27^.

The retrograde activation of the sinus venosus may propel blood away from the heart. In both anole lizard and python, we found venous valves immediately upstream of the sinus venosus that may prevent such backflow. These valves probably correspond to the venous valves encountered in the proximity of the heart in the subclavian and internal jugular veins of mammals^40^. Their position could allow for pressures to build up in the sinus venosus. In the pythons, we found that blood pressure of the sinus venosus increases after the ventricular systole of the previous beat and prior to the next atrial contraction, as previously reported for other species of snakes^35^. Further, we found that right atrial pressure increases in two steps with the first step coinciding with increments in sinus pressure, suggesting an active role of the sinus venosus in filling of the right atrium (Fig. 6). The sinus venosus also contributes to atrial filling in amphibians and in at least some fishes^1^. In some mammals, like bushbabies (*Galago*) and American opossums (*Didelphis*), the sinuatrial valve may persists between the inferior caval vein and the right atrium in a, presumably, competent state^41^ and their the sinus venarum may be well-equipped to generate pressure. The sinuatrial junction of most marsupial and placental mammals, however, appears to be so extensively remodeled that the bicuspid sinuatrial valve is rendered incompetent and there is an unguarded communication between the sinus venarum and the inferior caval vein^41^. During the extensive gestational remodeling of most mammals, the sinuatrial right leaflet will give rise to the Eustachian and Thebesian valves and remnants of the left leaflet form a fine meshwork on the interatrial septum or, more commonly, is absorbed altogether^13^.

Distally, the *Anolis* sinus venosus can be considered ‘atrialized’ because of expression of *Nkx2.5* and *Gja5*. The presence of Cx40 (*Gja5*) allows for fast impulse propagation but the electrical mapping did not reveal differences in propagation speed between parts of the sinus venosus. Propagation speed, however, also depends on the density of sodium channels which we did not assess in the anole lizard and python. Also, the myocardium of the reptile sinus venosus and atria respond similarly to adrenergic and cholinergic stimulation, as does the myocardium of the caval veins and atria of mammals^38, 42, 43^. The *Anolis* sinus venosus myocardium, however, in its entirety remains distinct from atrial myocardium by expressing *Tbx18*. Tbx18 expressed ectopically in the atria of the mouse decreases the levels of *Gja5* and *Scn5a* giving the atria a phenotype closer to that of the embryonic sinus venosus^44^. Also, the *Anolis* sinus venosus was the only chamber where *Tbx20* transcripts could not be detected, whereas in mouse, *Tbx20* is expressed in the entire heart, including the sinus venosus^45^ and is crucial for the developing and adult heart^46^. In the sinus venosus of reptiles, the significance of expression of *Tbx18* and absence of *Tbx20* may be that the sinus venosus is poorly atrialized. In the postnatal mammalian heart, atrialized sinus myocardium, like atrial myocardium, expresses gap junction proteins^47^, cardiac troponins^38^, and is regulated by sympathetic and parasympathetic agonists^42, 43^. Contractions of the atrialized sinus myocardium are in twitches and tetanus cannot be induced^42^. We show here that the atrialized parts of the sinus myocardium of reptile hearts have the same characteristics.

The myocardium of the caval veins of the mammalian heart is paradoxical, because it has no documented beneficial function but nonetheless poses a risk to cardiac function by exhibiting ectopic pacemaker activity^5, 42^. Current hypotheses on the function of the caval vein myocardium were originally formulated for the pulmonary venous myocardium; the systemic venous myocardium could function as a throttle valve inhibiting regurgitation from the right atrium to the veins, could aid right atrial filling, and could regulate systemic venous pressure and blood flow^42, 48^. The first and second function, however, are doubtful in light of evolution because the un-remodeled sinuatrial junction of the reptile heart has a valve, which prevents atrial regurgitation and the reptilian sinus venosus appears to aid atrial filling. For the sinus myocardium to substantially aid atrial filling there must be a sinuatrial delay, just as the atrioventricular delay provides time for the atria to aid ventricular filling, but the sinuatrial delay is present in reptiles and lost in mammals. It can then be postulated that the caval vein myocardium of the mammalian heart would be much better suited to fulfill its hypothesized functions, if it retained the reptilian configuration. So why the sinus venarum of the adult mammalian heart is invested with myocardium remains enigmatic.

The sinus myocardium of the adult mammal heart may simply be an unavoidable remnant of embryonic heart development. In modern bony fishes like the zebrafish, the sinus venosus forms with no myocardium, except on the sinuatrial junction where the dominant pacemaker is located, showing that myocardialization of the mesenchymal venous pole is not an inescapable process in vertebrate evolution^4, 5, 25^. Also, the embryonic arterial pole of reptiles, birds, and mammals is myocardial but it is incorporated to the ventricle in later stages and the adult arterial pole is essentially without myocardium^49^. Perhaps the simplest explanation for the presence of caval vein myocardium in mammals is that it is functionally beneficial in embryonic development, where the mammalian heart is much more reptile-like in form and function, and cannot be lost in later development.

Both reptiles and mammals form a so-called sinus septum, a myocardial ridge between the left and right sinus horns, which expresses *Tbx3* ^13, 50^. Derivatives of the sinus septum in human are prone to exhibit ectopic pacemaking^29^. In remodelling of the sinus venosus in mammals, the sinus septum seemingly contributes to the right atrial Eustachian ridge and the tendon of Todaro, key landmarks for the localization of the atrioventricular node^13, 51^.

In the monotreme heart, there is a sinus node^52^ and a well-developed sinuatrial valve^41^ that resembles the reptilian sinuatrial valve. It should be noted, however, that the electrical activity of the heart of monotreme mammals is only rudimentarily described^53^. Apparently, the monotreme heart does not undergo sinuatrial remodeling and it may be premature to state definitively that the sinuatrial junctional delay is lost in monotreme mammals.

This study reports the observation that the distal reptilian sinus venosus atrializes as revealed by the expression of genes otherwise exclusively found in the atria and ventricles, a trait that was thought to be specific of mammals. This implies that the specializations of the mammalian sinus venosus as compared to the assumed ancestral state represented by reptiles are principally the development of an anatomically identifiable sinus node and the loss of the sinuatrial delay.

## Materials and Methods

### Ethical statements

All experimental procedures on adult and embryonic material complied with national and institutional guidelines and were approved by Institutional Animal Care and Use Committee of the University of Amsterdam. The approval is registered as “DAE101617” for optical mapping of the anoles. All experiments were conducted prior to 2015, and prior to 2015 experiments in The Netherlands on non-mammalian embryos that are not autonomously viable did not require approval from the Institutional Animal Care and Use Committee. All experiments on pythons were carried out under the supervision of authorized investigators according to Danish Federal Regulations.

### Animals

Fertilized eggs of *Anolis sagrei* (N = 7) of Sanger stages 6–19^54^ were bought commercially. The specimens were dissected out of the shell and membranes and placed in phosphate-buffered physiological saline (0.9% NaCl) if video recordings were made of the beating heart and subsequently, or directly to 4% paraformaldehyde for 24 h if no recordings were made and then to 70% ethanol.

We bought commercially 8 *Anolis equestrie*, the largest species of the *Anolis*, for mapping of the electrical activation of the sinus venosus by electrode catheter (N = 3) and optical mapping (N = 5).

We bought commercially19 pythons (*Python regius*) of undetermined sex and age with body masses ranging from 69 g to 1376 g (458 ± 88 g; mean ± SEM). Prior to experiments the snakes were kept in vivariums at 30 °C with free access to water.

For localization of the pacemaker by immunohistochemistry we used, besides *Anolis* and *Python*, an embryonic Chinese softshell turtle (*Pelodiscus sinensis*, Tokita-Kuratani stage 17^55^), an embryonic *Varanus varius* (73 days post oviposition) as varanid lizards have higher heart rates (and maximal metabolism) than other reptiles^56^ and a near-hatching *Crocodylus palustris* (68 days post ovulation) and a Ferguson stage 16^57^ embryonic American alligator (*Alligator mississippiensis*) as crocodilians are the only reptiles with a full ventricular septum^9^.

### *In situ* study on anaesthetized snakes

A total of nine snakes were anaesthetized by an intramuscular injection of pentobarbital (30 mg kg^−1^, Sygehusapotekerne, Denmark) and mechanically ventilated with a Harvard Apparatus mechanical ventilator at 5–10 breaths min^−1^ and a tidal volume of approximately 60 ml kg^−1^. The heart was exposed by a 7 cm right ventrolateral incision. To measure blood pressures, occlusive PE50 catheters filled with heparinized saline (50 IU ml^−1^) were inserted in the vertebral artery (P_sys_) and in the sinus venosus via the right jugular vein (P_SV_). A flared catheter was inserted through a small incision in the right atrial lumen (P_RA_), the incision was closed with a tight suture. Electrocardiograms were recorded from 3 of the 9 snakes. All catheters were connected to disposable pressure transducers (Baxter Edward, model PX600, Irvine, CA) and the signals were amplified using an in-house built amplifier. The pressure transducers were calibrated against a static water column prior to each experiment and maintained at heart level. All pressure measures were recorded with a MP100 data acquisition system (Biopac Systems, Goleta, CA) at 200 Hz. No less than 30 min after catheterization injections of autonomic agonists were given in the vertebral artery in a volume of 1 ml kg^−1^ followed by a flush of approximately 0.2 ml kg^−1^ heparinized saline. After each agonist, the maximal response was compared to the pre-injection baseline. Agonists were given in the following order; adrenalin (5 µg kg^−1^), acetylcholine (2 µg kg^−1^, 22 mmol kg^−1^), isoproterenol (0.1 µmol kg^−1^), phenylephrine (10 µg kg^−1^). Hereafter we delivered blockade of muscarinic and β-adrenergic receptors with atropine (4 mg kg^−1^) and propranolol (4 mg kg^−1^) respectively. The efficacy of the blockade was verified with acetylcholine and adrenalin.

### *In vitro* studies of the isolated sinus venosus and the right atrium of python

Six pythons were deeply anaesthetized by pentobarbital (50 mg kg^−1^) and the hearts were excised and transferred to an ice-cold Ringer solution consisting of 95 mM NaCl, 25 mM NaHCO_3_, 2.5 mM KCl, 1 mM NaH_2_PO_4_, 1 mM MgSO4, 1.5 mM CaCl_2_, and 5 mM glucose with a pH of 7.5 then equilibrated with 2.0% CO_2_ and 98% O_2_ at 30 °C. One strip of sinus venosus and another of the right atrium were dissected out for each heart. Each strip was mounted vertically with surgical silk between a thin glass rod and a fixed platinum rod and placed in water-jacketed glass chamber containing 50 ml Ringer solution at 30 °C and equilibrated with a gas mixture of 98% O_2_ and 2% CO_2_ delivered by a Wosthoff pump. The glass rod was connected to a force transducer (Statham UC 2, Oxnard, CA, USA) to measure force of the isometric contractions. In a few instances with no spontaneous contraction, the atrial strips were electrically paced to verify viability. The strips were stretched to provide a near maximum twitch force. When the rate and force of the spontaneous contractions of the sinus venosus and right atrium had been recorded for a minimum of 30 min, a series of autonomic agonist were administered: Adrenaline (Adr, 10 µmol L^−1^), Acetylcholine (Ach, 1 µmol L^−1^), isoproterenol (Iso, 100 µmol L^−1^, with 500 µmol L^−1^ ascorbic acid), phenylephrine (Phe, 30 µmol L^−1^). When the maximal response had been recorded, the samples were washed twice for 5 min to remove any drugs. Hereafter a blockade of muscarinic receptors with atropine (Atr, 1 µmol L^−1^), and a subsequent blockade of β-adrenergic receptors with propranolol (Pro, 10 µmol L^−1^) were administered, no washing was performed after blockades, and these were left to settle for at least 10 min. The efficacy of the blockades were verified using acetylcholine and adrenalin.

### Electrode mapping of activation

We anaesthetized four pythons by an intramuscular injection of pentobarbital (30 mg kg^−1^, Sygehusapotekerne, Denmark) and ventilated at regular intervals through an intubation of the trachea. The heart was exposed by a 7 cm ventrolateral incision. Epicardial electrical activation was recorded using a CardioLab Electrophysiology Recording Systems (Version 6.5.6, GE Healthcare, US; amplifier by Prucka engineering, US). For the mapping an 8 polar 1.1 F catheter and a 4 polar 1.4 F catheter (1 mm electrode spacing, Millar Instruments, US) were used. Stimulations were performed with 5–10 mA in 2 ms (BioTek, US), where capture was observed in all chambers. Patterns of depolarisation in the ventricle, right atrium, and sinus venosus were recorded by the use of surface electrodes with a reference electrode fastened in a specific location on each chamber. In two snakes, the conduction velocities in atria and sinus venosus were derived with the P-wave of the surface electrograms as reference. To investigate the presence of decremental conduction (Wenckebach phenomenon), we used a programmed stimulation train of 3 cycles with an extrasystole in decreasing intervals down from 1800 ms^−1^ down to the Wenckebach point. The Wenckebach point of the sinuatrial and atrioventricular junctions were recorded by pacing the sinus venosus or the right atrium respectively, and before and after application of 5 µg kg^−1^ adrenaline into the vertebrate artery.

Cuban knight anoles (N = 3) were anaesthetized and ventilated as above for the pythons. Surface pads were placed on each limb to record ECGs. The sternum was then split and the pericardium cut away and the 8-electrode catheter was then placed on the sinus venosus for recording. The 8 electrodes were paired into 4 bipolar electrodes and the sinuatrial and atrioventricular delays were measured as the shortest delay recorded by any of the four electrodes. Propagation speeds were calculated from recordings that were preferably 6 mm (from bipolar electrodes 1 and 4), or 4 mm apart (from bipolar electrodes 1 and 3, or 2 and 4).

### Agonists and antagonists of the autonomic nervous system

All drugs were obtained from Sigma Aldrich (Denmark). All drugs were administered in 0.9% physiological saline and kept frozen after preparation. Prior to injection drugs were kept at 25–30 °C. To avoid oxidation of phenylephrine ascorbic acid was added (25 µmol kg^−1^ for a phenylephrine solution of 10 µg kg^−1^). Atropine, adrenaline, and phenylephrine were kept in foil to avoid light induced instability.

### Optical mapping of activation

The animals for optical mapping were decapitated and had the posterior caval vein catheterized with PE-90 immediately cranial to the liver. The catheter was filled with a custom made physiological saline and advanced to the sinus venosus after which the hearts were excised and transferred to the same physiological saline (in mM; NaCl 95, Tris 5, Glucose 5, KCl 2.5, CaCl_2_ 1.5, MgSO_4_ 1, pH adjusted 7.5 with acetic acid and NaOH).

The catheterized and excised hearts were incubated at 25 °C in the physiological saline 15 µmol/l di-4-ANEPPS (voltage sensitive dye). A 5-watt power LED (filtered 510 ± 20 nm) provided excitation light and fluorescence (filtered > 610 nm) was transmitted through a tandem lens system on CMOS sensor (100 × 100 elements; MICAM Ultima). Activation patterns were measured during sinus rhythm and optical action potentials were analyzed with custom software.

### Histology, *in-situ* hybridization, and immunohistochemistry

Specimens were embedded in paraffin and cut to series of 7–14 µm sections. Staining was with picro-sirius red (1 min differentiation in 0.01 M HCl), *in-situ* hybridization (as previously described^58^) or immunohistochemistry (as previously described^59^). For *in-situ* hybridization, we used previously described probes for *Anolis* mRNA (*Bmp2*, *Gja5*, *Tbx3*, *Tbx5*, *tnnt2* ^32^) and probes based on the following coordinates using UCSC Genome Browser on Lizard May 2010 (Broad AnoCar2.0/anoCar2) Assembly; *Hcn4* (chrUn_GL343517:230,977–269,752), *Isl1* (chr2:4,409,614–4,425,900), *Tbx18* (chr1:196,878,650–196,903,397), *Tbx20* (chr6:46249425–46294657)). Probes for the American alligator were made in house based on the following coordinates using UCSC Genome Browser on American alligator Aug. 2012 (allMis0.2/allMis1) Assembly: *Gja5* (JH733970:656,237–659,259), *Scn5a* (JH739807:162,160–168,550), *Tbx3* (JH733970:656,237–659,259). For visualization of Isl1 with immunohistochemistry we used a goat antibody to human Isl1 (Neuromics, dilution 1:200) visualized by binding to fluorescently labelled donkey-anti-goat antibody coupled to Alexa 680 (Invitrogen, dilution 1:250). Myocardium was visualized with a rabbit polyclonal antibody to human cardiac troponin I (Santa Cruz, dilution 1:400) visualized by binding to fluorescently labelled donkey-anti-rabbit antibody coupled to Alexa 488 (Invitrogen, dilution 1:250). All nuclei were stained with Dapi (Sigma, 1:40,000). We further tested antibodies for the proteins of several of the genes above, but we did not achieve specific detection. On the basis of section series we made 3D models using Amira® version 5.2 software of expression domains as described previously^60^.

## Electronic supplementary material

Supplementary Information
