# Supplementary material for: Morpho-functional characterization of the systemic venous pole of the reptile heart
Source: Sci Rep. 2017 Jul 27;7:6644. doi: 10.1038/s41598-017-06291-z (PMC5532247; doi:10.1038/s41598-017-06291-z)
Supplement: Supplementary file 1 — Supplementary Information [file 41598_2017_6291_MOESM1_ESM.pdf]

Supplementary figures

Morpho-functional characterization of the systemic venous pole of the reptile heart

Bjarke Jensen[1, *], Signe Vesterskov[2], Bastiaan J Boukens[1], Jan M Nielsen[3], Antoon FM Moorman[1], Vincent M Christoffels[1], Tobias Wang[2]

[1]*Department of Medical Biology, Academic Medical Center, University of Amsterdam, The Netherlands*

[2]*Department of Bioscience, Zoophysiology, Aarhus University, Denmark*

[3]*Department of Cardiology, Institute of Clinical Medicine, Aarhus University Hospital, Skejby, Aarhus, Denmark*

*Corresponding author: b.jensen@amc.uva.nl

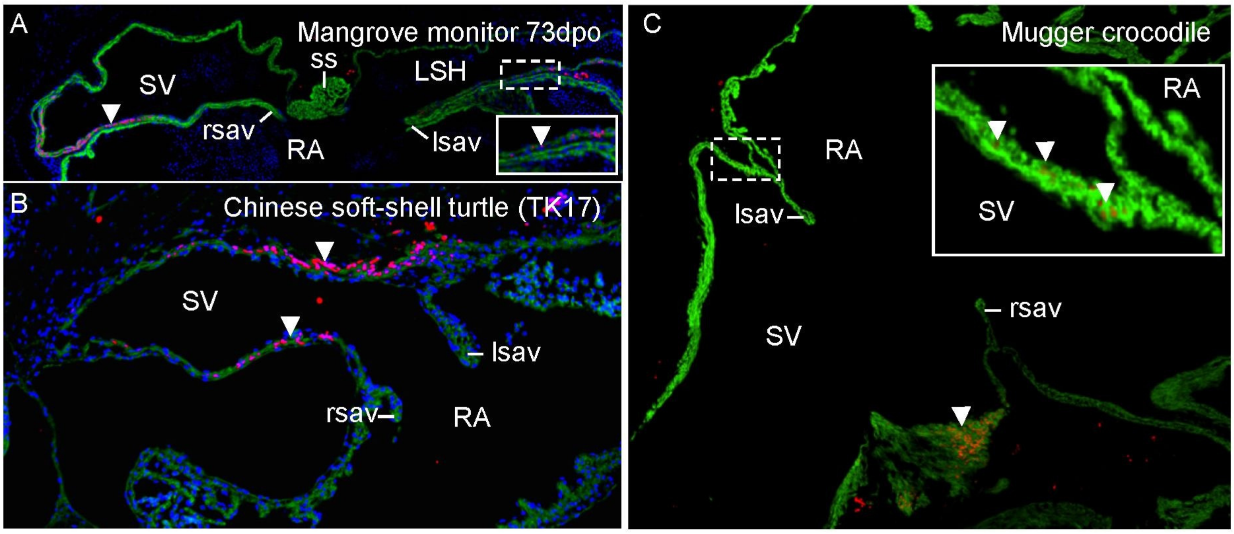

Fig. S1. Identification of the putative dominant pacemaker by Isl1 detection (arrowheads) in embryonic monitor lizard, turtle, and crocodile. A. Isl1 is expressed at the base of the sinuatrial valve leaflets in the embryonic mangrove monitor (A; 73/180 days post oviposition), Chinese soft-shell turtle (B; Tokita-Kuratani stage 17 47), and embryonic mugger crocodile (C; 68 days post oviposition). lsav, left leaflets of the sinuatrial valve; LSH, left sinus horn; RA, right atrium; rsav, right leaflets of the sinuatrial valve; ss, sinus septum; SV, sinus venosus.

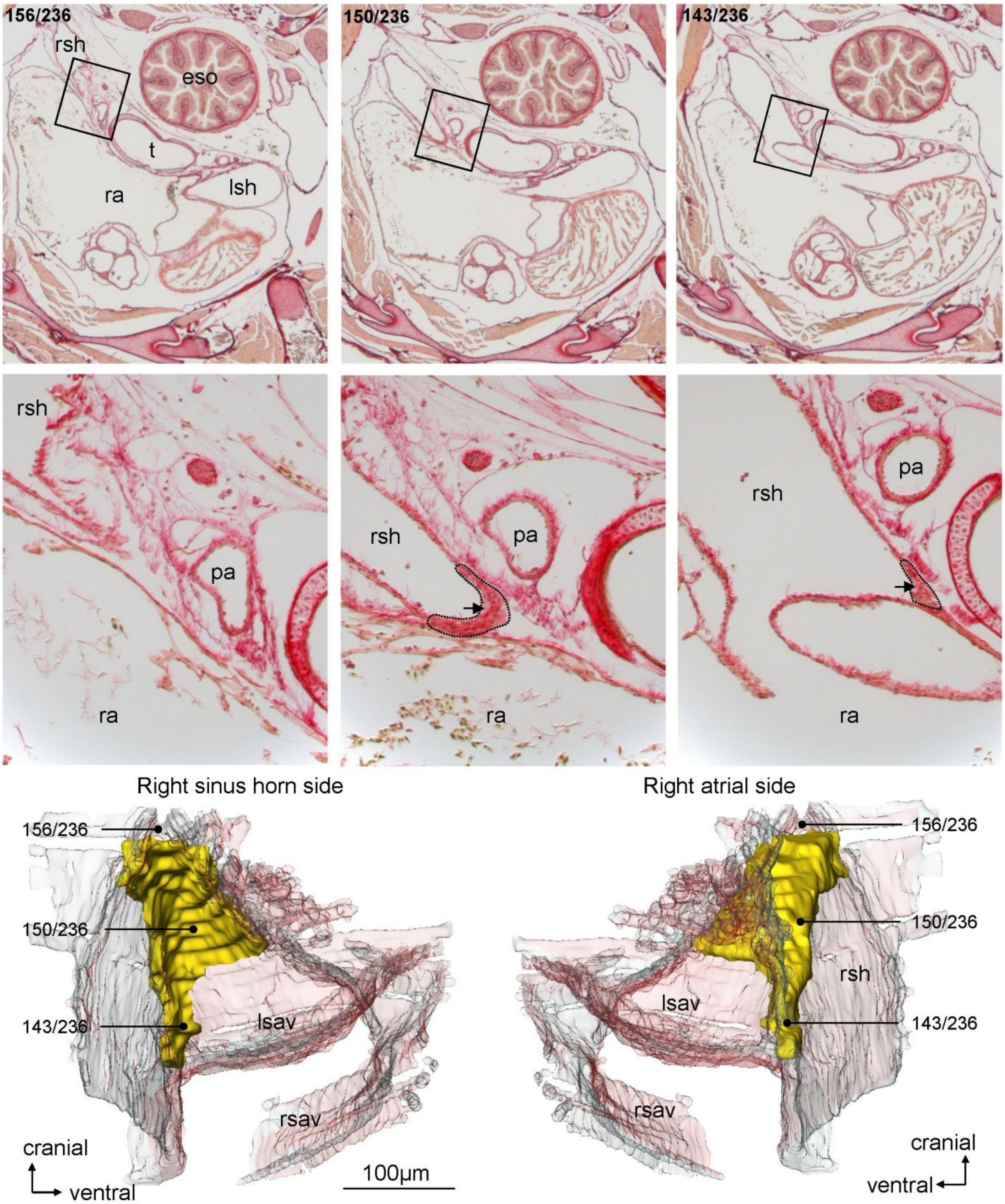

Fig. **S2**. In reptiles there is no insulated or otherwise anatomically identifiable sinus node, although the sinus myocardium is relatively thick in the cranial part of the sinuatrial junction, reconstructed in yellow. Images from a near-hatching brown anole. Numbers (xxx/236) indicate the section out of the 236 investigated sections. eso, esophagus; lsav, left leaflets of the sinuatrial valve; lsh, left sinus horn; pa, pulmonary artery; ra, right atrium; rsav, right leaflets of the sinuatrial valve; rsh, right sinus horn; t, trachea.

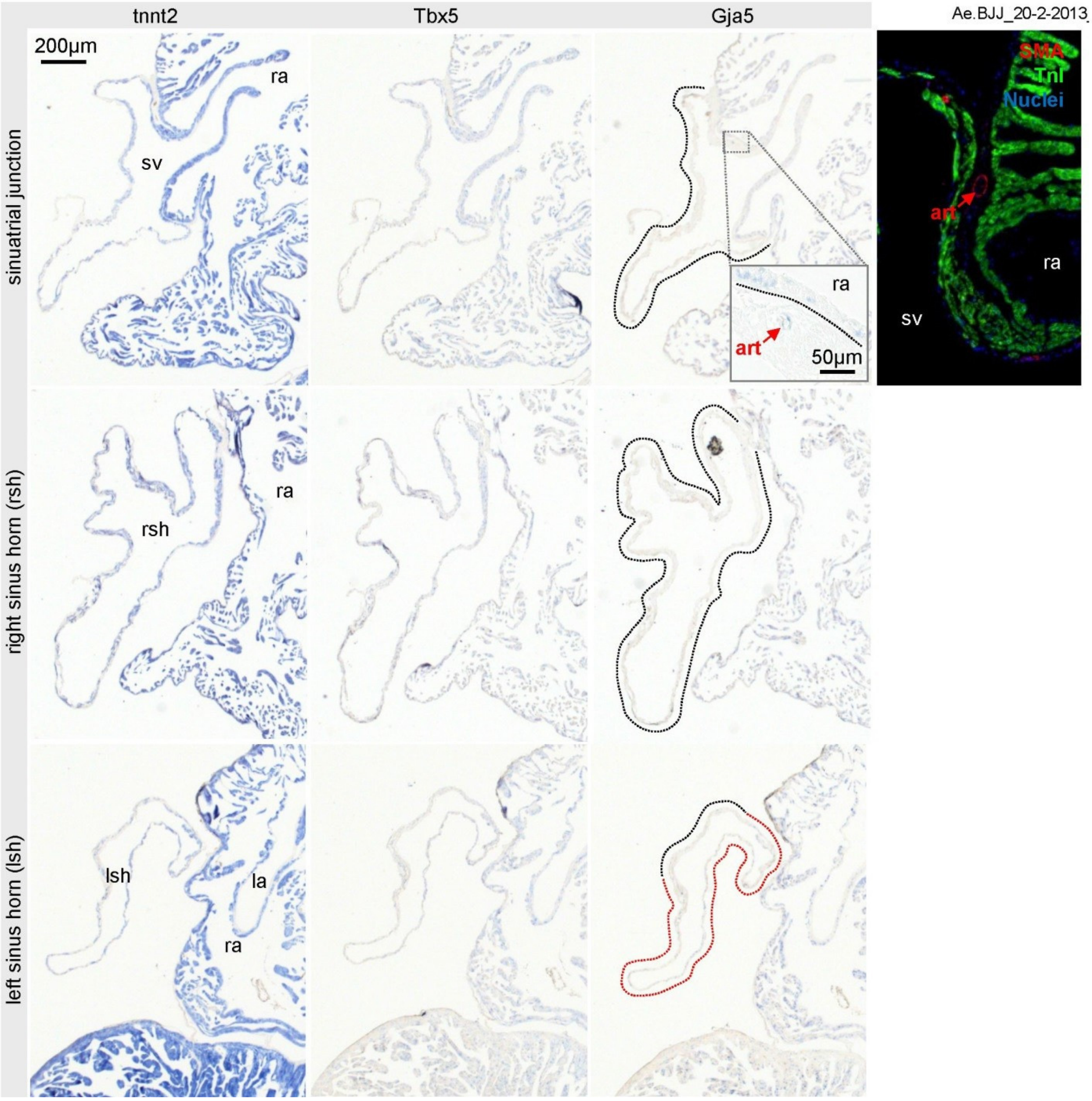

Fig. **S3**. The sinuatrial junction of reptiles may receive a relatively large coronary artery the endothelium of which expresses *Gja5* (red arrow) and the walls of which contains smooth muscle actin (SMA). The sinus myocardium proximal to the sinuatrial junction appears without Gja5 while distal parts of the sinus myocardium may express Gja5. Images from an adult knight anole. la, left atrium; lsh, left sinus horn; ra, right atrium; rsh, right sinus horn; sv, sinus venosus.

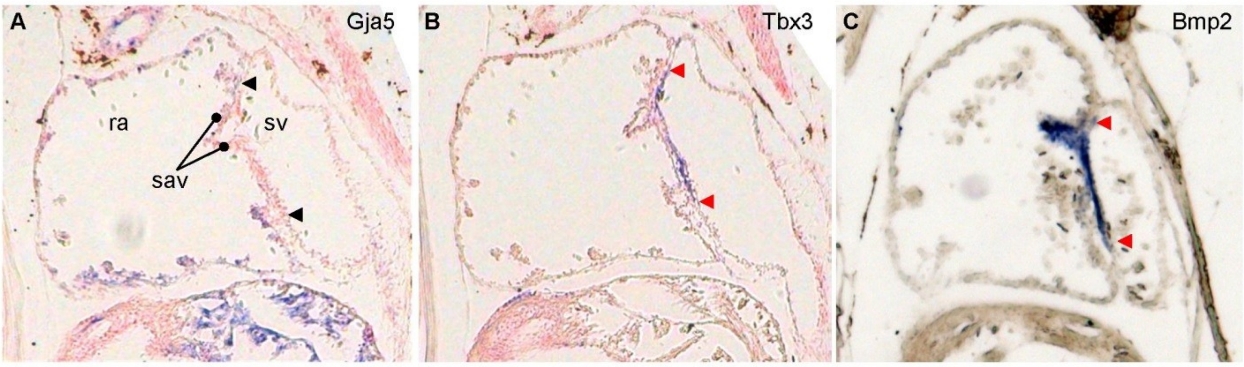

Fig. **S4**. The sinuatrial junction of reptiles expresses both *Tbx3* and *Bmp2* (red arrowheads indicates the margins of the expression domain). Images from a stage 16 brown anole. ra, right atrium; sav, sinuatrial valve; sv, sinus venosus.

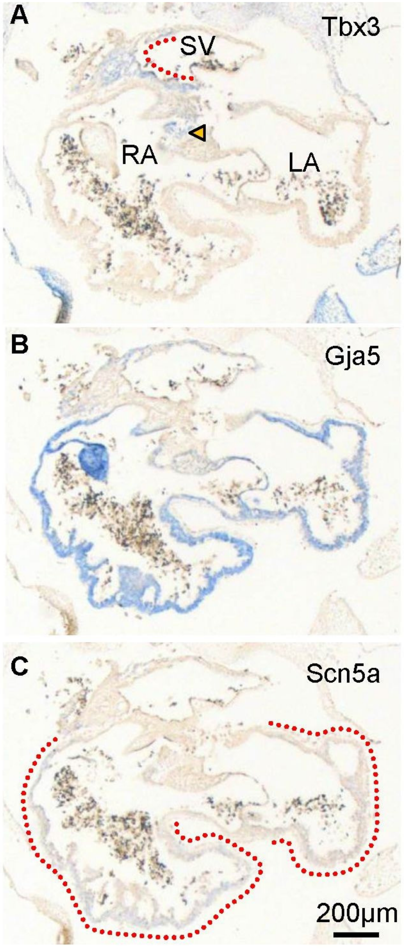

Fig. **S5**. The sinuatrial junction of the embryonic alligator (Ferguson stage 16) has the phenotype of the mammalian sinus node. A. *Tbx3* is expressed in sinus myocardium most proximal to the sinuatrial junction. The intra-atrial domain with *Tbx3* expression is the dorsal mesenchymal protrusion (orange arrowhead). B-C. The *Tbx3*-positive region appears without expression of *Gja5* and *Scn5a*. LA, left atrium; RA, right atrium; SV, sinus venosus.

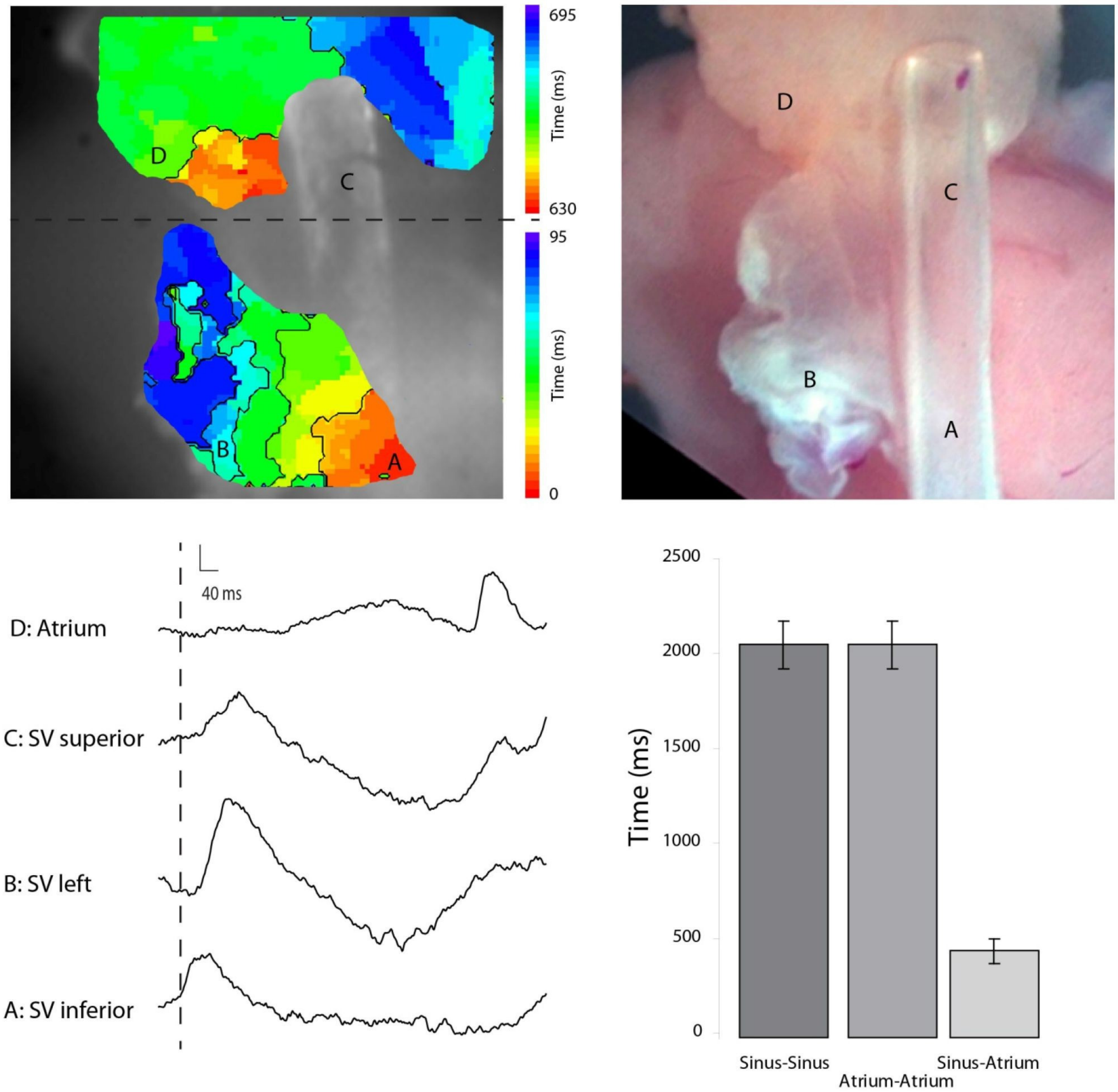

Fig. **S6**. Optical mapping of the excised heart of an adult knight anole, with a catheter inserted in the sinus venosus (C) to prevent collapse and excessive folding of the thin wall. Earliest activation was deep in the posterior sinus horn (A). The bar graph summarizes the intervals measured in 5 animals (average, error bars are standard deviation).

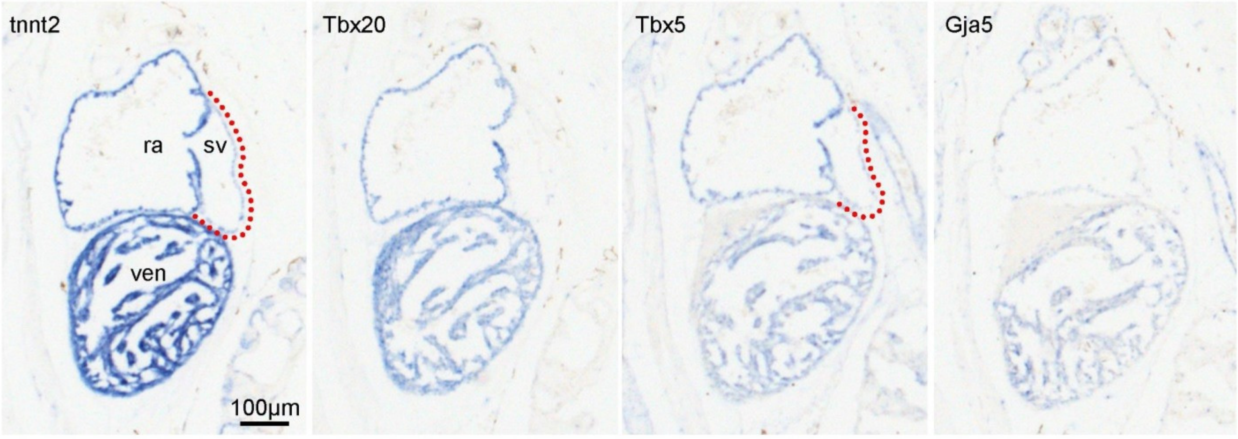

Fig. **S7**. The myocardium of the sinus venosus was molecularly distinct from the atria by the absence of Tbx20. Images from a stage 16 brown anole. ra, right atrium; sv, sinus venosus; ven, ventricle.

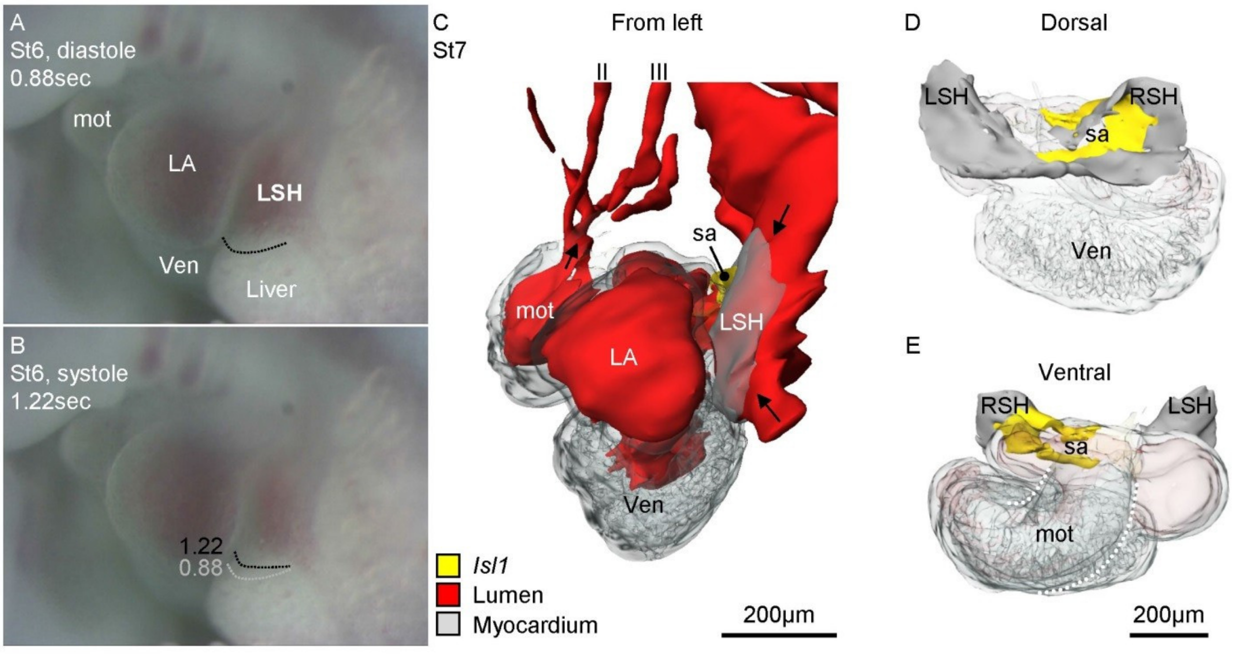

Fig. **S8**. Early in development, the left and right sinus horns express the myocardial marker tnnt2 and contract (A-B). Images from a stage 6 brown anole. C. Reconstruction of myocardium (transparent grey), blood (red) and myocardium expressing Isl1 (yellow) of the specimen of a stage 7 brown anole, oriented like the heart in A-B. D-E. Dorsal and ventral views showing the Isl1 expressing domain to be a substantial proportion of the sinus venosus, a proportion that diminishes in later development. II, second pharyngeal arch; III, third pharyngeal arch; LA, left atrium; LSH, left sinus horn; mot, myocardial outflow tract; RSH, right sinus horn; sa, sinuatrial orifice; Ven, ventricle.

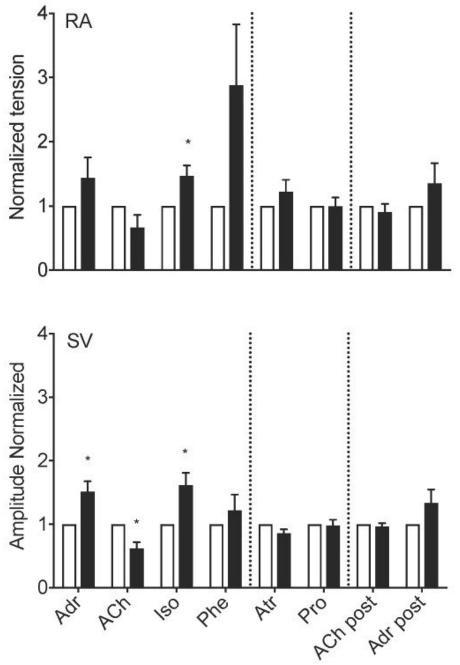

Fig. **S9**. Changes in tension in strips of sinus venosus (SV) and right atrium (RA) elicited by drugs related to effects of the central nervous system. *In vitro* amplitudes of spontaneous contractions are greater in strips of sinus venosus than strips of right atrium. Adr; adrenaline (10 µmol L^{-1}), ACh; Acetylcholine (1µmol L^{-1}), Iso; isoproterenol (100 µmol L^{-1}), Phe; phenylephrine (30 µmol L^{-1}). Atr; atropine (1µmol L^{-1}), Pro; propranolol (10 µmol L^{-1}) ACh post; acetylcholine after atropine, (1 µmol L^{-1}) and adrenaline after propranolol; Adr post; (10 µmol L^{-1}). N = 6. Asterisk indicates a significant difference from control value ($P < 0.05$, two-tailed, paired t-test).
